# Retinal toxicity secondary to subconjunctival cefuroxime following pars plana vitrectomy: A case report and literature review

**DOI:** 10.1016/j.ajoc.2022.101557

**Published:** 2022-05-02

**Authors:** Antony Raharja, James E. Neffendorf, Tom H. Williamson

**Affiliations:** St Thomas' Hospital, London, UK

**Keywords:** Cefuroxime toxicity, Retina, Endophthalmitis, Antibiotic prophylaxis, Pars plana vitrectomy, Post-operative complications

## Abstract

**Purpose:**

To present a case of inadvertent retinal toxicity induced by a standard dose of subconjunctival cefuroxime after epiretinal membrane surgery. Narrative review of cefuroxime overdose or toxicity after intraocular surgery was carried out to describe characteristics of cefuroxime toxicity and their relationship to visual outcome.

**Observations:**

A 64-year-old man underwent pars plana vitrectomy (PPV) with epiretinal membrane peel and received a standard dose of subconjunctival cefuroxime as endophthalmitis prophylaxis. At two weeks, visual acuity measured counting fingers, and fundus examination showed haemorrhages and cotton wool spots. Fluorescein angiography confirmed widespread ischaemia involving the macula. Subsequent litigation ruled that inadvertent cefuroxime toxicity after an accidental penetration of sclera was the likely aetiology.

**Conclusions and importance:**

In addition to inadvertent overdose due to dilution errors, accidental scleral penetration is another mechanism for drug toxicity following subconjunctival cefuroxime injection. Literature review revealed broadly different manifestations of cefuroxime retinal toxicity. This case highlights the need to consider severe cefuroxime toxicity in patients presenting with unexplained post-PPV visual loss.

## 1 Introduction

Antibiotics are routinely administered during intraocular surgery as prophylaxis against post-operative endophthalmitis. Previous case reports described retinal toxicity of varying severity after intracameral cefuroxime use in cataract surgery; this ranges from transient retinal oedema to severe haemorrhagic retinal infarction and optic atrophy.^1^, ^2^, ^3^, ^4^, ^5^, ^6^, ^7^, ^8^ A case series of 152 patients undergoing combined phacovitrectomy did not report any toxicity with intracameral cefuroxime.^9^

In vitreoretinal surgery, a more commonly used route of antibiotic delivery is via the subconjunctival space. Here, we report a case of retinal toxicity in a patient receiving subconjunctival cefuroxime 125 mg after pars plana vitrectomy (PPV).

## 2 Case report

A 64-year-old man with a preoperative best corrected visual acuity (BCVA) of 6/9 underwent epiretinal membrane (ERM) surgery in the left eye (LE) under general anaesthesia at a different institution. There was no significant past medical history or previous ocular surgery. PPV using 23-gauge sclerotomies with routine ERM peel and a fluid fill was performed. At the end of the procedure, subconjunctival cefuroxime (125 mg in 1 ml) was given for endophthalmitis prophylaxis, although volume delivered was not documented. The procedure was reported to be technically uneventful, although it was immediately complicated by inferior subconjunctival haemorrhage and a vitreous cavity haemorrhage was noted on the following day.

**Fig. 1:**
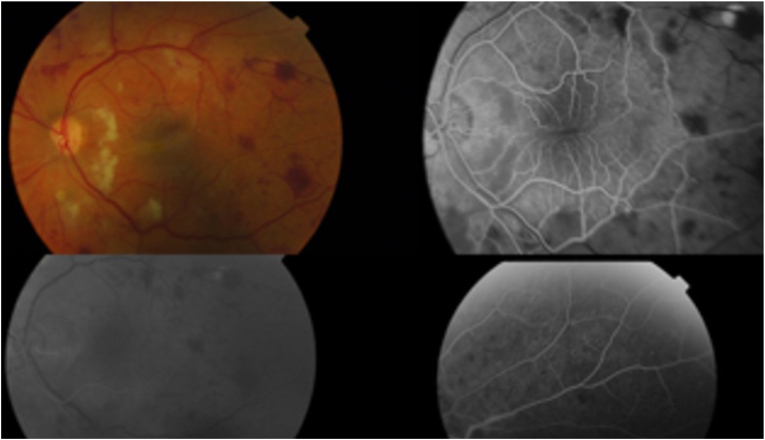
Fundus photograph two weeks post-operatively showed widespread deep retinal haemorrhages and cotton wool spots, more prominent in the midperipheral and peripapillary areas. Fundus fluorescein angiography shows widespread diffuse leakage and ischaemia.

BCVA declined to finger counting at day one and this persisted despite resolution of vitreous haemorrhage. The intraocular pressure (IOP) at day two was 14 mmHg. At two weeks post-surgery, the patient sought a second opinion with our service. Slit lamp examination revealed subconjunctival haemorrhage, clear cornea, IOP 11 mmHg, deep and quiet anterior chamber, clear lens and no vitreous inflammation. Fundus examination showed macular fold. In addition, there were widespread deep retinal haemorrhages and cotton wool spots more prominent in the midperipheral and peripapillary areas. There was no vascular tortuosity and the optic disc was not swollen. Fundus fluorescein angiogram showed widespread leakage and ischaemia (Fig. 1). BCVA worsened to light perception due to the maculopathy, and a macula involving retinal detachment subsequently developed within four months of the original procedure.

He underwent PPV with silicon oil tamponade, subsequent retinectomy due to proliferative vitreoretinopathy and lastly, a silicon oil removal. There were no pre-operative or intra-operative findings such as a retinal tear, needle marks on the retinal pigment epithelium or choroid, or choroidal haemorrhage which might have confirmed scleral penetration by the subconjunctival injection during the initial vitrectomy. Despite a flat retina, in the absence of tamponade, the LE BCVA did not improve. The retina at five-year follow-up was largely white and ischaemic, with only a small island of healthy retina. Final visual acuity measured light perception.

A litigation was raised against the surgeon who performed the ERM peel procedure. As clinical features resembled severe cefuroxime toxicity, the court ruled that an accidental penetration of the sclera during subconjunctival injection was the likely aetiology. Another potential mechanism considered was increased intraocular exposure to cefuroxime through a leaking sclerotomy.

## 3 Discussion

Our case identifies accidental penetration of sclera as a potential mechanism of cefuroxime toxicity after subconjunctival administration following PPV. Other factors that may theoretically increase the risk of toxicity include small eyes, sclerotomy leak and the use of intraocular tamponade.^10^ This case highlights the need to consider cefuroxime toxicity in patients presenting with unexplained post-PPV visual loss.

We reviewed 102 other published cases of intraocular cefuroxime toxicity or overdose; all but one involved intracameral cefuroxime administration following phacoemulsification cataract surgery (Table 1).^1^, ^2^, ^3^, ^4^, ^5^, ^6^, ^7^, ^8^^,^^11^, ^12^, ^13^, ^14^, ^15^, ^16^, ^17^ The majority of overdose cases were related to dilution or dosing errors during the preparation of intracameral cefuroxime.^1^, ^2^, ^3^, ^4^, ^5^, ^6^, ^7^, ^8^ There were 33 cases of toxicity in patients apparently receiving a standard recommended intracameral dose of 0.1 ml of 10 mg/ml solution, although the administered volume was not specified in several reports.^11^, ^12^, ^13^, ^14^^,^^16^^,^^17^

**Table 1:** Summary characteristics of 103 cases of cefuroxime overdose or toxicity after intraocular surgery.

Author (year)	n/N	Dose (mg)	Mechanism of overdose	PCR ± AV	Non-retinal manifestations	Retinal manifestations	Treatment	BCVA at final review
**Overdose without toxicity**								
Sakarya, 2010^2^	0/6	3	Dilution error	No	None	None	N/A	6/6
**Transient macular serous detachment and/or oedema**								
Faure, 2015^17^	1/1	1	N/A	No	None	Transient SMD with schisis-like appearance at ONL	None	6/6
Aslankurt, 2016^11^	8/8	1	N/A	No	None	Transient SMD and intraretinal fluid	None	Median 6/7.5^+2^
Chlasta-Twardzik, 2020^13^	1/1	1	N/A	No	None	Transient SMD with macular oedema predominantly in the ONL	None	6/6
Xiao, 2015^14^	2/2	1	N/A	No	None	Transient SMD and macular oedema predominantly in the ONL	None	6/6
Zuo, 2018^12^	20/20	1	N/A	No	Mild corneal oedema and very mild AC inflammation	serous neurosensory retinal detachment and macular oedema	None	Mean 6/7.5
Buyukyildi^1^ (2010)	2/2	2	Dilution error	No	Trace AC cells	large SMD with intraretinal fluid accumulation in the outer retinal layers	Case 1: systemic acetazolamide and steroids. Case 2: IVTA	Case 1: 6/6
								Case 2: 6/7.5
Wong, 2015^3^	6/13	9	Dilution error	No	2/13 (15%) mild central corneal oedema. Mild AC inflammation	6/13 (46%) transient macular oedema resolving within one week	None	Mean 6/7.5
Delyfer 2011^5^	6/6	30–50	Dilution error	No	2/6 (33%) Corneal oedema, AC inflammation, vitritis	Transient large SMD, macular oedema predominantly in ONL	None	6/9.5 in one case; 6/7.5 in others
						FFA: diffuse leakage, normal retinal perfusion, no macular ischaemia		
Kontos, 2013^15^	1/1	31.2 SC^a^	Standard subconjunctival dose	No	Minimal AC inflammation Minimal vitritis	Neurosensory macular detachment with cystoid macular oedema	Oral flubiprofen	6/9.5
						FFA: mild patchy choroidal filling, no leakage from macular capillaries		
**Retinal haemorrhagic infarct**								
Sul, 2018^16^	1/1	1	N/A	Yes	Corneal oedema and vitritis	Extensive retinal haemorrhage and later optic atrophy	Systemic steroids	Counting finger
						OCT: Foveal thinning, OS atrophy		
						FFA: leakage and capillary infarct		
Qureshi, 2011^6^	1/1	62.5	Subconjunctival preparation given intracamerally	No	Corneal oedema	Haemorrhage and mild tortuosity	Immediate washout and IVTA	3/60
						FFA: ischaemic macula and late dye leakage at week 2		
Cifti, 2013^7^	4/4	50–70	Not reported	Yes	Corneal oedema	Widespread haemorrhages and optic atrophy	None	Light perception or hand movement
Current case	1/1	Up to 125 SC^a^	Standard subconjunctival dose but scleral penetration	No	None at two weeks	Widespread haemorrhages and peripapillary cotton wool spots	None	Light perception
						FFA: widespread leakage and ischaemia		
**Other reported manifestations of cefuroxime toxicity**								
Kamal-Salah 2019^4^	2/5	10	Dilution error	No	None	1/5 (20%) ellipsoid layer disruption	None	In patients with ellipsoid layer disruption, 6/12, 6/18, 6/18, 6/24, 6/36, 6/60. In others, 6/6
						1/5 (20%) transient SMD with intraretinal oedema		
	6/14	12.5	Dilution error	No	1/14 (7.1%) AC inflammation and vitritis	5/14 (36%) ellipsoid layer disruption	None	
						1/14 (7.1%) subjective colour alteration		
Olavi, 2012^8^	16/16	10–250	Dilution error	No	Corneal oedema and loss of corneal endothelial cells	Retinal pigmentary changes	None	Four patients have poorer post-operative BCVA (worse than 6/30)

n: number of affected patients; N: total number of patients in the case series; PCR ± AV: Posterior capsule rupture with or without anterior vitrectomy; BCVA: Best corrected visual acuity; OCT: optical coherence tomography; SMD: serous macular detachment; FFA: fundus fluorescein angiogram; ERG: electroretinogram; IVTA: intravitreal triamcinolone.

a Indicates subconjunctival (SC) route of administration.

There were six severe cases with poor visual outcomes (worse than 6/60) demonstrating early retinal haemorrhages with or without macular ischaemia and optic atrophy.^6^^,^^7^^,^^16^ These occurred with accidental very high doses, with the exception of one case in which a standard dose was apparently used. For the latter, cataract surgery was complicated with posterior capsule rupture (PCR) and anterior vitrectomy.^16^ Cefuroxime toxicity in all cases involving PCR and anterior vitrectomy resulted in poor visual outcomes.^7^^,^^16^

There is currently no proven treatment for severe cefuroxime toxicity. Systemic or intravitreal corticosteroids were used in two cases.^6^^,^^16^ Immediate washout was attempted in one case perioperatively.^6^ However, visual outcomes in these cases were 3/60 or worse despite treatment (Table 1).

Cases with good visual outcomes displayed early transient serous macular detachment (SMD) associated with intraretinal oedema predominantly in the outer nuclear layers.^1^^,^^3^, ^4^, ^5^^,^^11^, ^12^, ^13^, ^14^, ^15^^,^^17^ This developed rapidly (within 24 hours) but quickly resolved within one to two weeks without treatment. One case series attributed this to the practice of inflating clear corneal incisions with cefuroxime/balance salt solution (BSS) mixture.^11^ Ellipsoid layer disruption was an observed feature in one case series involving six patients with variable visual outcomes (ranging from 6/12 to 6/60), but was not reported in other cases.^4^

Our case is in keeping with the more severe manifestations of cefuroxime toxicity. This is in contrast with the only other report of subconjunctival cefuroxime retinal toxicity following a dose of 31.25mg.^15^ In this case, the patient experienced SMD and macular oedema which resolved by day six. This may suggest variability and risk of toxic intraocular concentrations after subconjunctival cefuroxime injection and, as illustrated by our case, its potential to reach a toxic level after an accidental scleral penetration. Other factors affecting intraocular drug concentration after subconjunctival delivery include volume injected and in the context of PPV, the presence of sclerotomy leak, intraocular tamponade and ocular size.^10^ This may support using commercially prepared intracameral cefuroxime formulations as endophthalmitis prophylaxis for pars plana vitrectomy to provide a more consistent intraocular concentration with a lower risk of toxicity. It is important though to note that intracameral cefuroxime does carry a risk of toxicity if a dilution error occurs.

## Patient consent

Consent to publish the case report was not obtained. This report does not contain any personal information that could lead to the identification of the patient.

## Funding

This study was funded by Eyehope (UK registered charity 1119866).

## Authorship

All authors attest that they meet the current ICMJE criteria for Authorship.

## Declaration of competing interest

The following authors have no financial disclosures: AR, JN, TW.
